# Telehealth and digital developments in society that persons 75 years and older in European countries have been part of: a scoping review

**DOI:** 10.1186/s12913-021-07154-0

**Published:** 2021-10-26

**Authors:** Moonika Raja, Jorunn Bjerkan, Ingjerd G. Kymre, Kathleen T. Galvin, Lisbeth Uhrenfeldt

**Affiliations:** 1 Faculty of Nursing and Health Sciences, Nord University, Bodø, Norway; 2 Faculty of Nursing and Health Sciences, Nord University, Levanger, Norway; 3 School of Health Sciences, University of Brighton, Brighton, United Kingdom; 4 Danish Centre of Systematic Reviews, a Joanna Briggs Institute Centre of Excellence, Centre of Clinical Guidelines, Aalborg University, Aalborg, Denmark

**Keywords:** Telehealth, Digital devices, Health services for the aged, Societal digital demands, Aged 80 and over

## Abstract

**Background:**

Demographic changes are leading to an ageing population in Europe. People are becoming more dependent on digital technologies and health ministries invest increasingly in digitalisation. Societal digital demands impact older people and learning to use new telehealth systems and digital devices are seen as a means of securing their needs.

**Methods:**

The present study undertakes a scoping review in order to map relevant evidence about telehealth and digital developments in society involving citizens aged 75 and over in European countries. It focuses on their experiences and the main barriers to, and facilitators of, societal digital demands. A framework proposed by Arksey and O`Malley was used to guide the scoping review process. The studies included in the review covered telehealth, digital technology and digital devices, and the context covered participants` own home or surroundings. A comprehensive search on PubMed/MEDLINE, CINAHL, Scopus, Embase and Open Grey was undertaken.

**Results:**

Out of 727 identified citations, 13 sources which met the inclusion criteria (9 original study articles, 2 theses, 1 letter about a product and 1 project report). Few of the studies identified have investigated European citizens 75 years and older separately. The studies included varied in their design, location and focus. Older people have experienced both telehealth and digital devices making life easier and the opposite. The outstanding facilitator found was that technology should be easy to use, and difficulty in remembering the instructions was seen as an important barrier. Interestingly, both social support and lack of social support were found as facilitators of using new devices.

**Conclusions:**

Telehealth may give a sense of security but learning to use a new device often takes extra effort. Older people were more open to new devices if the possible advantages of the new technology outweighed the effort that would be involved in adopting a new strategy. As technology develops rapidly, and life expectancy in Europe is anticipated to rise continually, there is a need for new and additional research among older European citizens. Future research should cover the technical solutions most relevant to older people today, social support and participants` access to the devices.

## Background

The world is facing an ageing population and the proportion of older people is expected to grow even more [1]. The number of people aged 75 years and older in European countries is projected to expand by 60.5 % by 2050 [2]. The growing number of older people within society poses a range of challenges, creating a significant impact on socio-economic structures and providing a stimulus for the development of new goods and telehealth services adapted to the needs of the older generation [2, 3].

Digital technologies are steadily transforming our world and changing our daily life [4, 5]. As much as any other age-group, digitalisation impacts older people [6]. Older adults are a highly heterogenous group with differing needs and they require specific technological and telehealth solutions [7]. Societal digital demands see learning to use new technologies as a means of securing older people`s health needs and human rights [8, 9].

The European Union`s Charter of Fundamental Rights [10] declares that human dignity is inviolable and must be respected and protected. Dignity is the affirmation of something valuable in oneself or another; in its variations a gathering of both common vulnerability and common value [11]. Dignity has a wide range of protective functions as well as having reciprocal, relational and social aspects [12]. The loss of dignity is especially noticed in its rupture [11]. As telehealth and digital devices can give older people more autonomy, it may impact their dignity in both positive and negative ways [13, 14]. Policy in Europe is moving in the direction of addressing the issue. The European Commission [6] underlines that in shaping Europe`s digital future it is very important that every citizen reap the benefit of an increasingly digitalised healthcare and society.

Research from the beginning of the 21st century shows that the proportion of older people using telehealth and information and communication technologies (ICT) is low. In this period, an example from Scotland about ICT use in healthcare among older people, suggests that satisfied clients tend to be under 80 years [15]. Another example, a survey related to telehealth conducted in 15 European countries among older people, showed that the respondents interest in various telehealth systems declined considerably with age [16]. Later research in Europe includes a wider range of different types of telehealth and digital technology, such as smartphone apps, wearable devices and robotics. Digital development is rapid, and the list continually expanding [17]. European studies from the last 5 years also claim an age-related digital divide: a recent study from the United Kingdom found that age 65 years and older was the strongest inverse correlate of using physical activity surveillance through wearable trackers [18]. Likewise, research among people with hypertension in Austria and Germany showed that age has a negative association with the intention to use mobile health applications [19]. Literature, generally, shows that as telehealth and ICT evolves, seniors are open to using it, but there are interface barriers such as lack of knowledge and confidence, costs, health-related obstacles and lack of guidance in the use of new digital devices [20, 21].

Continuous digital development brings wider use of home-based telemedicine [22]. The term telemedicine, coined in the 1970 s, refers to the use of ICT to improve patient outcomes by increasing access to care and medical information [23]. Technological developments led to a newer and wider term, telehealth, which refers to a broader scope of remote healthcare services [24]. According to WHO, telemedicine and telehealth both comprise 4 elements: (1) the purpose is to provide clinical support, (2) it is intended to overcome geographical barriers, (3) it involves the use of various types of ICT and (4) its goal is to improve health outcomes [24]. As continual digital development has brought even more new terms and definitions, a European Parliamentary Technology Assessment reports on the usage of the wider term eHealth covering telehealth, telecare, telemedicine, tele coaching and mHealth [3]. WHO defines eHealth as a secure and cost-effective use of ICT in support of health and health-related fields. Furthermore, eHealth can be seen as the use of modern ICT to meet the needs of citizens, healthcare professionals and patients by improving prevention, diagnosis, treatment, monitoring and management of health and lifestyle [28]. According to WHO are terms telehealth and eHealth both in use but in this study, due to including historical perspective, will hereinafter be used term telehealth.

Digital technology is a wide term that in addition to telehealth covers all electronical tools, technological devices and automatic systems that generate, store or process information [6]. Digitalisation concerns bringing together people, data, and processes; it is something that affects human experience [3]. For purposes of this review our focus was on telehealth but some digital technology engagement of note did emerge in the review.

Older people have experienced that telehealth and digital technology has the potential to assist them in remaining independent [14]. Usage of telehealth may benefit citizens with the complex, multidimensional problems many older people suffer from [25]. It can improve quality of life for homebound older people and increase the amount of time they can live independently outside of an institution [13]. A review study about facilitators of, and barriers to, the adoption of telehealth in persons older than 65 years found that the use of telehealth among older adults is expected to rise, but in order for effective adoption, it is important to keep the patient`s perspective at the forefront [26]. A systematic review covering worldwide studies of ICT among older citizens, published in English between 2002 and 2015, suggested that ICT could be an effective tool to tackle social isolation among older people. However, it is not suitable for every senior alike [27].

Digital demands impact health ministries to invest increasingly in telehealth and digitalisation [3, 28]. The use of ICT and homebased telehealth brings new facilitators but new barriers as well [22]. Existing reviews from Europe covering older people, telehealth and technology include only certain groups of older citizens such as those in palliative care [29] or patients suffering from Alzheimer`s disease [30]. A review from 2013 covers findings specifically related to telehealth applications for people aged 55 and over [31]. As societal digital demands develop rapidly, up-to-date research is needed [22]. A recent systematic review of ICT solutions included studies covering a wider range of older people, but only involved ICT solutions that have been implemented or deployed in pilot form contributing to the key smart ageing and excluding research materials on telemonitoring and telehealth programmes which include self-monitoring [32]. Reviews from 2011 to 2020 underline that the true needs of older people as end-users are poorly known and further research is needed in order to utilise future ICT solutions. Furthermore, Arief et al. [31] emphasised that the barriers of using telehealth can be overcome by utilising the facilitators. Munn et al. [33] claim that scoping reviews can be used when the purpose of the review is to identify types of available evidence, identify knowledge gaps and clarify concepts.

In this scoping review, we considered papers that included a population 75 years and older at the time of the study. The statutory pension age in Europe is between 60 and 67 years [2] which must be set against a growing demand from all sectors for employees with basic digital skills, and adults who are actively working have experienced more or less digitalisation in connection with their work [6]. As 75 years marks about 10 years from their transition into retirement, this age-group has spent recent years further and further from the labour force digital transition and has not experienced digitalisation in connection with their work in the way younger adults have. The concept of included studies covered telehealth, digital technology and digital devices. From 1st January 1998, a whole new period in Europe`s transition to the Information Society, with the complete liberalisation of all telecommunications networks and services in the European Union, began [34]. In this study, the context was Europe, and participants` own home or home surroundings. In order to utilise future telehealth and ICT adapted to the needs of older citizens, one must determine whether existing solutions are satisfying their needs.

The aim of this scoping review was to map a body of literature, summarise and discuss research findings concerning historical telehealth and digital development over the last 20 years that people 75 years and older in European countries have been part of. Moreover, to identify research gaps in the existing literature in order to inform future research.

This review was guided by a broad main research question:

1. *What is known from the literature about what citizens 75 years and older in European countries have experienced, as society has developed digitally (1998-2018)?*

Furthermore, two secondary research questions provided structure to this review:

2. *What are the main barriers for people 75 years and older in European countries concerning societal digital demands?*
3. *What are the main facilitators for people 75 years and older in European countries concerning societal digital demands?*

## Method

### Protocol

The scoping review protocol was developed by the researchers in collaboration with a university librarian. The a priori peer-reviewed protocol was followed throughout the process [35]. It is described briefly below.

The framework proposed by Arksey and O`Malley [36] was used to guide this scoping review process. The original framework has been further developed by Levac and colleagues [37] and the Joanna Briggs Institute [38]. The review process included 5 stages: (a) identifying the research questions; (b) identifying relevant studies; (c) selecting studies; (d) charting the data and (e) collating, summarising and reporting the results [36].

### Eligibility criteria

The criteria for inclusion in the scoping review was primary research studies with different study designs: qualitative studies, quantitative research and mixed method research. As recommended by the Johanna Briggs Institute [38], unpublished literature was included. According to Paez [39], grey literature search may be an invaluable component of a review and may include theses and dissertations, research and committee reports, government reports, and ongoing research, among others. Text (e.g., government recommendations and political documents) and opinion papers were also included. Papers published between 1998 and 2018 were considered. This period was chosen because digital development has spread rapidly during the last two decades [17, 5]. Selected publications had to include only persons 75 years and older that live at home or include separate results from this population. From these, publications that included telehealth, digital devices and communication technology in European countries were selected. Articles in Danish, English, Estonian, Finnish, German, Norwegian, Russian and Swedish were considered for inclusion in this review in order to include non-English research [40].

### Information sources and search

A controlled vocabulary and key word search was conducted using the following electronic databases: Embase, CINAHL, MEDLINE via PubMed, Scopus and Open Grey. The search strategies were drafted by the researchers in collaboration with a university librarian. The keywords used during the search are shown in Table 1.

**Table 1:** Keywords used during the search

	Population	Concept	Context
Keywords:	Aged	Communication technology	Europe
	Elderly	Digital assistant	Home-based
	Nonagenarian (90-99yrs)	Digital demands	Homecare
	Octogenarian (80-89yrs)	Digitalization	Home care
	Older	eHealth	Home setting
	Oldest old (80yrs+)	Electronic health	Outside
	Senior	Health informatics	Own home
	75	Health technology	
		mHealth	
		Mobile Health	
		Telehealth	
		Telemedicine	
		Technology	
Headings:	Aged	Computer Communications	Europe
	Aged, 80 and over	Digitizers/ES	Home care
	Aging Geriatric	Networks Assistive technology	Home health care
		Technology/ES	
		Telecommunications	
		Telecommuting	
		Telemedicine	
MeSH terms:	Aged (65-79yrs)	Telemedicine	Europe
	Aged, 80 and over		Home care services (by professional)
			Home health nursing
			Home nursing (by family)

Keywords not covered by the protocol were added during the search process. In the strategy, we used English search terms. The specific terms changed slightly depending on the database. However, the main keywords were used throughout. Boolean logic containing combinations of MeSH Terms and Text Words was used [41]. The final search strategy for MEDLINE can be found in Table 2. The final search results were exported into EndNote, and duplicates removed. Reference lists of included articles were visually scanned to ascertain whether any key studies had been missed.

**Table 2:** MEDLINE Search Strategy via Pubmed

#	Searches	Results
1	Aged OR elderly OR oldest old OR octogenarian OR nonagenarian	5 434 075
2	Telemedicine OR Mobile Health OR mHealth OR telehealth OR eHealth OR digital assistant	71 142
3	Home care OR home care services OR home nursing OR home health nursing OR own home	827 013
4	Europe	1 482 707
5	#1 AND #2 AND #3 AND #4	602
6	Limit to 1998/01/01- 2018/12/31	513
7	Limit to Danish, English, Estonian, Finnish, German, Norwegian, Russian, Swedish languages	475

### Selection of sources of evidence

**Fig. 1:**
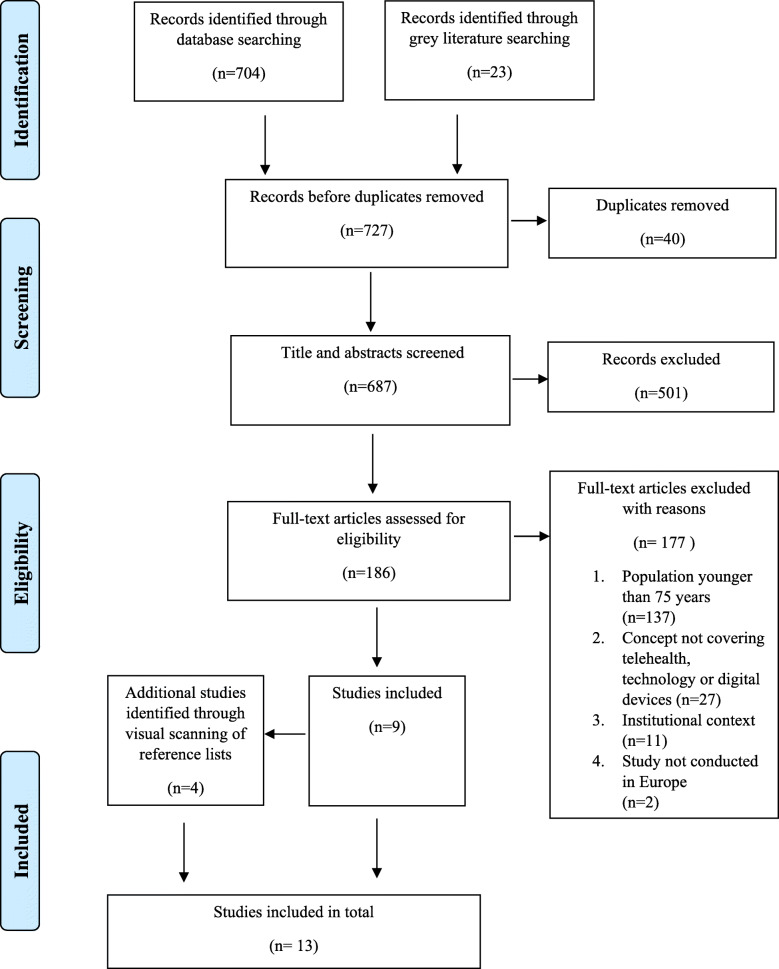
PRISMA flow diagram illustrating the search strategy^1^. ^1^This flow diagram provides the phases of article identification and selection, which resulted in the identification of 13 references that were deemed eligible for inclusion in the review. Prepared in accordance with Tricco et al. [63]

The inclusion and exclusion criteria were tested on a sample of abstracts (*n*=50) before conducting the search. No changes were made. We included studies according to inclusion and exclusion criteria through a two-step process: a title and abstract review and full-text review (see PRISMA flow diagram, Fig. 1), following the protocol [35]. The literature search results were retrieved from each database and imported into a reference management software. After removing duplicates, all abstracts were screened by two authors. Then, all potentially relevant full articles were reviewed by the first and second author. Reference lists of potential articles were visually scanned to ascertain whether any key studies had been missed. Potential articles were reviewed by the first and second author. The degree of agreement at full-text review was 98,4 %, discrepancies were resolved in discussion with the third and fifth author and then all potential articles were agreed for inclusion by all authors.

### Data charting process and data items

We used standardised data collection forms developed by the research team for this study [35]. The first author tested the form with 3 sources. After testing, definition of study population was added to the data collection form. The information abstracted included: authors, title, journal, year of publication, country, study population, setting, methodology, aim of the study, type of technology/telehealth/digital demands described, reported outcomes, facilitators of, and barriers to the use of digital technology. First author charted the data, and the other researchers verified the data for accuracy. For obtaining missing data, the author of 1 study was contacted. The information gathered from the selected sources was organised into tables to reflect the objectives of this study (outcomes about what older people have experienced, the main barriers and the main facilitators of using different types of digital devices and technology). The tables contained information about type of characteristics and the results. The final version of the tables is presented in the results section (see Tables 3, 4, 5 and 6). We have discussed, compared, and contrasted the findings of different studies in the results section following the tables.

## Results

### Selection of sources of evidence

In this review, 727 abstracts were retrieved, and after deduplication 687 abstracts were reviewed. A total of 186 publications were selected from a title and abstract review for a full-text review. Among the excluded studies, common reasons for exclusion included population younger than 75 years (*n*=137), concept not covering telehealth, technology or digital devices (*n*=27), hospital or institutional context (*n*=11) and studies not being conducted in Europe (*n*=2). A total of 10 publications satisfied the inclusion criteria, 2 of the included articles were about the same study, but research methods and outcomes were different. Of 10 sources, 1 article was excluded because the study and methods were similar to those described in another included article [42]. Reference lists of the articles included were visually scanned to ascertain whether any key studies had been missed: 4 additional publications were found this way. The total number of papers to review was 13, see PRISMA flow diagram (Fig. 1). An additional search was conducted in October 2020 to find any potential new sources from 1st January 2019 to up to date. There were 179 abstracts retrieved. Through following the PRISMA strategy, none of the found sources met the inclusion criteria, a common reason for exclusion was population younger than 75 years.

### Characteristics of sources of evidence

Of the 13 studies selected for the final analysis, 2 were conducted in 3 European countries (Germany, Finland, Italy or Austria, France, Hungary) [43, 44], 6 were conducted in Sweden [45–50], 2 in Estonia [51, 52], 1 in Finland [53], 1 in Germany [54] and 1 was conducted in Italy [55] (see Tables 3 and 4). There were 5 articles including only a population 75 years and older [45, 50, 53–55] and 8 studies included also other groups (significant others of the participants) [43, 44, 46–49, 51, 52], but the participants were divided into age-groups and the results of each group were given separately, which made it possible to study the results of those 75 years and older.

The sample size of participants 75 years and older, varied between 1 and 1007. In addition, 1 study gave information about contacts made to a medical helpline [45] but there was no information about how many times each individual had made the call. However, 7477 contacts were made. All the studies included were published between 1998 and 2018, but 2 studies used information collected earlier in the 90 s: 1 of them started a pilot in 1991 [54], the other collected data in 1995 [43]. Of the 13 included sources, 9 articles were published in international journals [43–50, 54], 2 were theses [51, 52], 1 was a letter about a product [55] and 1 was a project report [53]. There were 10 studies in English [43–50, 54, 55], 2 in Estonian [51, 52] and 1 in Finnish [53]. Of the analysed studies, 5 explored telehealth [45, 46, 49, 50, 54], 2 robots [44, 55] and 6 other types of technology [43, 47, 48, 51–53].

The studies` population, concept and context are described in Tables 3 and 4. The context of these studies was homecare [46–50, 54], home surroundings [45, 47, 52, 53], test-centre-based [44, 51, 55] and one had an outdoor environment [43]. There were 11 qualitative [44, 46–55] and 2 quantitative studies [43, 45]. Of the 13 included studies, 5 explored the usage of new digital technologies provided to the participants in trials lasting between 20 days and 7 years [46, 48–50, 54], 2 studies explored the general usage of technological solutions in daily life [47, 53], and 1 in the outdoor environment [43]. Furthermore, 1 looked into calls made to the medical helpline during 1 year [45], 1 explored the usage of smartphones [52], 1 looked into the usage of the internet [51] and 2 studies introduced a new device to the participants and asked their opinion about it [44, 55]. As 46 % of the included studies were conducted in Sweden, the focus it shows in this country will be discussed separately. Table 3 gives information about characteristics of sources from different European countries and Table 4 gives information about studies conducted in Sweden.

**Table 3:** Characteristics of sources of evidence from different European countries

Author(s) and year	Population	Concept	Context
Stroetmann and Erkert 1999 [54]	17 mobility-impaired older people (aged 75 to over 90)	Videophone service (HausTeleDienst). The clients were connected to the service centre using a standard cable-TV network	Homecare in Germany
Marcellini et al. 2000 [43]	1007 older people with different health conditions (aged 75 years and older). In addition, younger age-group	Ticket machines, automatic teller machine and phones that use phone cards	Outdoor environment in Germany, Finland and Italy
Perle 2012 [51]	75-year-old lady. In addition, 20-year-old lady	The internet	Test-centre in Estonia
Wessmann et al. 2013 [53]	22 older people (aged between 75 and 90 years)	Technology used by older people (for example computer, phone), information technology	Home surroundings in Finland
Zsiga et al. 2013 [44]	11 older people (aged between 77 and 85 years). In addition, caregivers	Robot. The functions demonstrated included speech recognition, navigation, reminder function, shopping list creation and video conferencing	Test-centre in Austria, France and Hungary
La Tona et al. 2017 [55]	4 older people presenting a wide range of age-related disorders (aged between 87 and 89 years)	Robot with voice, gesture and touch interfaces.	Test-centre in Italy
Adamsoo 2018 [52]	5 older people (aged between 75 and 81 years). In addition, younger age-group	Smartphone	Home setting in Estonia

**Table 4:** Characteristics of sources of evidence from Sweden

Author(s) and year	Population	Concept	Context
Magnusson and Hanson 2005 [46]	5 older people with neurological disorders (aged between 75 and 83 years). In addition, spouses	ICT based support service for older people and their family carers that consists of a range of multimedia caring programs that the family access via their personal computers	Homecare in Sweden
Nygård and Starkhammar 2007 [47]	3 older people with dementia (aged 76, 82 and 75 years). In addition, younger age-group	Everyday digital technology (televisions, electronic machines, remote controls, cell phones and computers)	Home setting in Sweden
Rosenberg and Nygård 2011 [48]	3 older people with dementia (aged between 79 and 91 years). In addition, significant others	An electronic calendar, a speaking clock and a digital watch with six built-in alarms	Homecare in Sweden
Lind and Karlsson 2014 [49]	7 older people with diagnosed heart failure(aged 75 years and older). In addition, spouses	Digital pen-and-paper technology with a digital IR camera that records everything the pen writes, and data can be transferred to a server via Internet	Homecare in Sweden
Lind et al. 2016 [50]	14 older people with diagnosed heart failure (aged 75 years and older)	Digital pen-and-paper technology with a digital IR camera that records everything the pen writes, and data can be transferred to a server via Internet	Homecare in Sweden
Dahlgren et al. 2017 [45]	Older people (aged 80 years and over). 7477 contacts were made but there is no information about if each individual was calling more than once	Nationwide medical helpline, Healthcare Guide 1177 by Phone in Sweden	Home setting in Sweden

### Results of individual sources of evidence

The studies` aims and reported outcomes together with factors that made using digital devices easier or harder can be found in Tables 5 and 6. Table 5 represents results of individual sources of evidence from different European countries and Table 6 represents results from Sweden.

**Table 5:** Results of individual sources of evidence from different European countries

Study	Aim of the study	Reported outcomes	Facilitators	Barriers
Stroetmann and Erkert 1999 [54]	To provide the capability for frail older people to live independently	The system was rated positively, impact on quality of life of the participants	Feeling of being in power over technology. Easy-to-use remote- control unit. Possibility for direct help, if needed	The system was not completely reliable. Technical problems
Marcellini et al. 2000 [43]	To examine the use of ticket machines, automatic teller machines and telephone cards by older people	The use of these technologies is low. Age is most important predictor factor of using the machines. Younger participants feel that such technology makes life easier	Technology makes life easier. Both social support and lack of social support. Easy to use technology	Technology makes life more difficult. Low education
Perle 2012 [51]	To compare the habits of older people and young people in using internet	Older persons had more specific problems (related to insufficient knowledge of the structure of the web and computer) than younger	Experienced older person teach other older people. User-friendly web-design. Repeated practice gives skills and confidence.	Knowledge is insufficient. No appropriate training. Teachers are too quick. Difficulties with new systems.
Wessmann et al. 2013 [53]	To investigate how older people experience the use of technology	No link was found between technology and quality of life. Age was related to the use of technology. There was a clear link between technology usage and level of education	High education. Younger age. More experience with technology. Financial benefit Safety factor Communication with family and friends	Low education. No need to use technology. Expensive. Need assistants when use it. Functional limitations due to age
Zsiga et al. 2013 [44]	To collect the opinions of the participants about the robot	Robot has potential to be useful for older individuals. Communication with the robot should be better. The robot helps to compensate memory loss	Gain a companion. Robot providing physical help in tasks. Robot sending an alarm signal. Providing reminder function. Video connection. Companionship.	Robot`s camera might not respect their private life. The design of the device. It cannot replace people. Hard to understand.
La Tona et al. 2017 [55]	To validate the developed interfaces of robot	Good users` feeling towards the interfaces. The voice interference and alarm were found useful, while web pages and touch-screens commands were less appreciated	People can be monitored. Robot makes people more autonomous. Robot can monitor room and gives vocal feedback	Touchscreens are difficult to use. Difficulties remembering the commands. Robots appearance is unnatural
Adamsoo 2018 [52]	To find out what are older peoples` main concerns about using smartphones	Coping with digital devices is significantly affected by English language skills, high screen sensitivity, small text size, clumsiness of hands.	Technology gives a sense of security, contact with family, independence, helps to reach help. Help from family about how to use the device. The device is practical	No need for innovations. Memory difficulties. Device is too fast, small or expensive. Inaccurate motor skills. Little practice

**Table 6:** Results of individual sources of evidence from Sweden

Study	Aim of the study	Reported outcomes	Facilitators	Barriers
Magnusson and Hanson 2005 [46]	To see whether the use of ICT systems will reduce the potential use of other services	Cost savings were achieved. Quality of life of the participants had increased through using the service	Contact with call centre enables feeling more secure. Contact with other families. Programs help to feel less anxious. Possibility to learn	It is not comparable with benefits of nursing home. Difficulties using technology
Nygård and Starkhammar 2007 [47]	To identify difficulties in the use of technology by persons with dementia	Difficulties with encompassing conditions that interfere with the use of the technology, limitations in the use of instructions and deficiencies in the communication between users and technology	Help from family. Practicing in home environment. Writing introductions down	Memory deficits. Deficits in attending to multiple aspects. Sensitivity to stress. Appearance of the devises
Rosenberg and Nygård 2011 [48]	To capture the experiences of bringing assistive technology into the life of person with dementia	Right placement of the device is important. Support could facilitate the use of the device in itself but user`s maneuverability became so limited that using the device was no longer meaningful. Persons needed time to try out the devices	Understanding how to use the devices. The device has a meaning for the user. Support in using the device. Personal motivation	Using a new device is burdensome. The possible advantages did not outweigh the effort that would be involved. Difficulties remembering the instructions
Lind and Karlsson 2014 [49]	To describe the experiences in using telehealth system	The digital pen was found easy to use, it gave a sense of security. Even the “digitally illiterate” may use the Internet	Easy to handle. Helps to keep track of the patient`s symptoms. Feeling being closer to the clinic. Getting help when using it	Technology is “a bit scary”. No previous experience using similar technology. Not able to handle the system by themselves.
Lind et al. 2016 [50]	To report experiences of the implementation of a home telehealth system	System was easy to use and method saved time. The participants felt more secured and involved in their own care. The system helped to prevent hospitalisation	It saved time. Easy to contact clinicians. Gave sense of security and closeness to the clinic	Being too weak to use the system by himself. The tool not working
Dahlgren et al. 2017 [45]	To describe contact made by older people to Sweden`s nationwide medical helpline	The utilisation rate of the service by older people was high. Women had higher incidence rate. The most common reason for contact was drug-related questions	Possibility to get answers to health- related questions. Possibility for administrative procedures (e.g. requesting a copy of their medical record)	Not discussed in the article

### Synthesis of results

In accordance with the inclusion criteria, studies in the review included older peoples` experiences with reference to different types of telehealth and other digital technology. Digital devices used in the studies included telehealth systems such as digital pen and cable-tv-based videophone service, assistive devices like electronic calendars, speaking clock, digital watch, homecare robot, and other technology like smartphone, computers, ticket machines, automatic tellers and telephones that using telephone cards (see Tables 3 and 4). As the digital devices were very different, the experiences and outcomes also varied. Synthesis of results were conducted based on research questions by dividing the results into 3 categories. Below, the results for Sweden and the other European countries are presented separately in those categories: outcomes about what older people have experienced, the main barriers and the main facilitators of using different types of digital devices and technology.

1. *What is known from the literature about what citizens 75 years and older in European countries have experienced, as society has developed digitally (1998-2018)?*

#### Technology has impact on older people`s lives

Older people in Sweden experienced that the services had a positive impact on their lives. Participants who used a digital-pen telehealth system or ICT based support services felt more secure [46, 50]. Older people`s experience of using an ICT support service showed that the system had the potential to reduce the use of other services whilst maintaining their quality of life [46]. Experience of using assistive devices in Sweden showed that these tools had the potential to be useful, but persons might need time to try out the devices [48]. At the same time, it was found that digital devices might need to be adapted or combined with something else in order to be beneficial [48]. Rosenberg and Nygård [48] concluded that digital devices might make some older participants` lives easier; however, others might feel the contrary. Older people in Sweden experienced that using a new digital technology could at first be frightening or that extra effort was required to adopt a new strategy. Digital-pen users generally found the technology “*a bit scary*” but using a telehealth system did not frighten them [49]. Users of digital assistive devices in Sweden felt that adopting a new strategy needed extra effort [48].

Similarly to Sweden, older people from Germany experienced that telehealth systems had a positive impact on their lives. Participants using specially developed videophones between home and residential care centre stated that they felt less lonely and had more joy in life [54]. In contrast, a study from Finland, that investigated how older people experienced the use of technology, found no link between technology and quality of life, but found that technology could bring a new kind of comfort [53]. Results about exploring homecare robot operating suggested that robots had the potential to be useful for older individuals but could not replace people [44].

#### Technology is making life easier and the opposite

In parallel to Sweden, older people in other European countries experienced both technology making life easier and the opposite. Some older people in Germany, Finland and Italy described their experience of using ticket machines, automatic teller machines and telephones in an outdoor environment as making life easier, while some felt the opposite [43]. As with older people in Sweden, participants in other European countries experienced that using a new digital technology could be frightening at first or that extra efforts were needed to adopt a new strategy. Zsiga and colleagues [44] discovered that homecare robots frightened older people, but not more so than any other new technology. Using a telehealth system in Germany unnerved participants because, in the beginning, they thought that the system was not completely reliable [54]. Smartphone users in Estonia felt that adopting a new strategy needed extra effort [52].

2. *What are the main barriers for people 75 years and older in European countries concerning societal digital demands?*

#### Difficulties using telehealth and other technology

The results regarding the use of telehealth systems in Sweden found that the main barriers for older people could be being physically too weak to use the device by themselves or the tool not working properly [50]. ICT based support service users in Sweden also claimed that they had some difficulties in using the technology [46]. Other barriers were that the design of the devices limited the opportunities of placement due to electric cabling, and that using a new device was too burdensome [48]. Functional limitations due to age, including difficulties remembering the instructions, were pointed to as barriers by older people in Sweden [47, 48].

As with participants from Sweden, it was found among telehealth users in Germany and internet users in Estonia that technical problems might be seen as a barrier [51, 54]. Older people in Finland said that they often needed assistance when using technology, but that they did not want to depend on others [53]. The appearance of the device was seen as an important barrier by older people in Austria, Hungary and Italy when considering using robots [44, 55]. Functional limitations due to age, including difficulty remembering the instructions, were pointed to as barriers by older citizens in Finland commenting about technology, participants using ambient intelligence architectures in Italy and smartphone users in Estonia [52, 53, 55]. Another barrier found by Marcellini and colleagues [43] was participants` personal opinions, that technological solutions made life more difficult, which led to less use.

#### Concerns about privacy and the link between use of technology and education

Older people considering using homecare robots were concerned that the robot`s camera might not respect their privacy [44]. Conversely, Stroetmann and Erkert [54] found that older people have far less fear of being observed than researchers expected. In 2 studies, a link was found between education and technology usage. One study concluded that older people with lower education used less technology in general, and another study found that they used less automated technology in an outdoor environment [43, 53]. Other included studies did not contain information about technology and education.

3. *What are the main facilitators for people 75 years and older in European countries concerning societal digital demands?*

#### Technology gives a sense of security

In several studies, participants from Sweden mentioned that technology gave them a sense of security because it helped them to reach assistance [46, 49, 50]. Telehealth systems connected older people to medical workers [45, 46, 49, 50]. In Sweden, it was found that both social support and lack of social support facilitated the use of digital assistive devices [48]. Support from spouses when using a digital-pen system was also stated as being helpful [49]. Participants who believed that digital assistive devices would be really meaningful adopted them more easily [48].

As in Sweden, older people in other European counties stated that the opportunity to get in contact with someone, when using technology, was motivating. Homecare robots helped to start video connection with family members [44, 55], and telehealth systems gave an opportunity to get in contact with medical workers [54]. Further, older people from Estonia, Finland, Germany and Italy stated that technology gave them a sense of security because it helped to reach assistance [44, 52–54]. In Finland, Germany and Italy, it was discovered that both social support and lack of social support facilitated the use of automated technology [43]. An Estonian study found that if one of the spouses was an experienced smartphone user, this was helpful for the other spouse [52].

#### Personal positive opinion about the digital device could facilitate use of technology

A personal positive opinion about the digital device could also be seen as a facilitator. Older people who were using more automated technology in an outdoor environment stated that such technology made life easier [54]. Adamsoo [52] found that older people in Estonia were interested in using smartphones if they found them to be useful.

## Discussion

The purpose of this scoping review was to map a body of literature and summarise research findings concerning historical telehealth and digital development, focusing on the main barriers to, and facilitators of, societal digital demands and experiences over the last 20 years that people 75 years and older in European countries have been part of. Moreover, the goal was to identify research gaps in the existing literature in order to inform future research.

The findings of this review suggest that only a few of the identified studies have investigated European citizens 75 years and older separately. A majority of the studies meeting the inclusion criteria for concept and context were excluded because there were no separate results for that population. Of the 13 articles included just 5 focused only on citizens 75 years and older, 8 articles included other groups (such as spouses, younger age-group and caregivers) as well, but the results were given separately. As Europe is facing an ageing population, and as this provides a stimulus for developing new goods and telehealth services adapted to the needs of older people [1–3], it is essential to investigate older European citizens` experiences and needs in a digitally led society. This study confirms the findings from Nymberg et al. [56], that information from older people about their needs in telehealth interventions is important in order for successful implementation.

Of the 13 included studies, 6 were conducted in Sweden. Most likely, this can be explained by the Nordic countries being positioned as digital front-runners in a European context [57]. Strong research environments aimed at promoting research both into telehealth and ageing may help Sweden to stand out among other Nordic countries [58]. The Swedish Government has stated that digital skills are in their priority areas, and that the development of telehealth and ICTs supporting older people is vital [3, 57]. That gives us the possibility of learning from the Nordic countries. However, only 2 of the 6 studies included and conducted in Sweden had their focus solely on citizens 75 years and older.

The findings of this scoping review show that the most important facilitator for older people using telehealth and digital devices is that technology should be easy to use: 9 out of 13 studies (69 %) stated this as being paramount. Interestingly, both social support and lack of social support were stated as facilitators of using new technology, including telehealth. Having someone next to you to help with obstacles while using technology was important for some older citizens, whilst lack of social support made lonely older people try out technology because they had no one else to do it for them. Other facilitators found, using telehealth and ICT, were the opportunity to get in touch with someone, new technology saving time, the digital device looking nice and having a meaning for the user. These results are in agreement with O`Connor et al. [59] that found individuals considering several different quality aspects of a digital health service before signing up to it. Older people in Sweden and other European countries have experienced that telehealth systems had a positive impact on their lives, but some digital devices might need to be adapted or combined with something else in order to be beneficial. Participants experienced both telehealth making life easier and the opposite. These findings suggest that telehealth and ICT usage by older people has different pathways and is in accordance with Peek et al. [60] who found that technology acquisition by seniors may be characterised as a heterogenous process.

Mantovani and Turnheim [61] stated that, in Europe, older people are expected to embrace technological shifts just as much as other age-groups. The findings of this scoping review suggest that there are likely to be a range of barriers needing to be overcome if we want older people to use and benefit from telehealth and technological shifts. It was mentioned several times that functional limitations due to age, including difficulties remembering the instructions, were seen as an important barrier [47, 48, 52, 53]. It is also essential to ensure that the devices respect older persons` privacy, that usage would not need too much effort from people who are already fragile, and the design of the devices should not limit the opportunities of placement. The appearance of the devices is equally important, and the possible advantage of the new system should outweigh the effort involved in adopting a new strategy. Other barriers included telehealth not properly working, the system not being completely reliable and difficulties understanding the device. It is in accordance with research about telehealth adoption in 24 European countries that reports about the lack of technological skills among patients to understand the devices [62].Included studies gave no information about use of telehealth and ICT impacting older people`s dignity.

The review found limited evidence that use of technology was connected to level of education, even though this was emphasised as a barrier in 2 of the studies. Earlier experience of using technology was also covered slightly in 2 studies, and little experience was not seen as an important barrier. The finding that earlier experience of using technology and level of education were not seen as dominant “push” facilitators to the use of new digital solutions might help to open a door for learning for those older people who would not dare to try telehealth and new devices because of a lack of previous experience.

As telehealth and ICT develops rapidly, additional and new research is required. The studies included in this review covered different types of telehealth, digital devices and technology. Some of devices in these studies are no longer in use as new modern technologies have replaced cable-tv-based telehealth videophone services, telephones that use telephone cards are barely seen and other of the technologies described might have been changed or upgraded. New telehealth systems and technical solutions are being developed constantly [3, 5]. Future research should cover the telehealth systems most relevant to this population today, but there is still potential to learn from previous studies. Fresh telehealth systems and technological solutions are always new for the user trying them for the first time, just as older solutions once used to be brand new. The learning patterns have similarities that can provide us with needed information. Technology acquisition by older people has many different pathways [60] and further research is necessary.

As the studies included gave little information about educational level and the usage of telehealth systems, this needs further investigation. Also, issues such as earlier experience using technology and access to the devices could be covered by future research. As both social support and the lack of social support have been seen as important facilitators of the adoption of new telehealth systems and other technology, they deserve attention in future research. In addition, qualitative studies could focus on what kind of social support is needed in order to facilitate the uptake of new telehealth systems. As life expectancy in Europe is expected to rise continually [2], there is a need for new and additional research concerning older European citizens.

## Strengths and limitations

Using a comprehensive, systematic search strategy based on a priori peer reviewed protocol to identify a diverse range of studies with different designs, was a strength of this review. It was a strength that articles in 8 European languages were considered and sources in 3 languages were included to the final analysis. Furthermore, it should be acknowledged that unpublished literature was added and the data analysis process of this review followed Arksey and O` Malley`s suggestions [36]. Furthermore, this scoping review covered 20 years from January 1st 1998 to December 21st 2018, and additional search was conducted in October 2020, to see if there were any new sources meeting the inclusion criteria. However, corresponding limitations included the selection of studies including only persons 75 years and older or studies where separate results were given for that age-group. Findings from papers covering wider groups of older people with a mean age of 75 years or older may have provided additional insights into the range of barriers and facilitators concerning societal digital demands in citizens 75 years and older in European countries. Of the 13 included studies, 4 were identified through visual scanning of reference lists. Our search covered keywords only in English but 3 of the studies did not comprise English keywords and 1 covered keywords not used in our search strategy (distributed ambient intelligence, IoT robotics, modular user interface and smart buildings).

## Conclusions

Older people in Europe have experienced telehealth and ICT both as making life easier and simultaneously the opposite. It may give a sense of security but learning to use a new device often takes extra effort. There should be a balance between the potential benefits and the effort required. It was found that older people were more open to new devices if the possible advantage of the new technology outweighed the effort that would be involved in adopting a new strategy. A majority of the studies (*n*=9; 69 %) stated the importance of telehealth and ICT being easy to use. The appearance of the devices was also important. As social support and lack of social support were both seen as important “push” facilitators of adapting telehealth systems and new digital devices, “push” and “pull” facilitators deserve closer attention in future research. The findings of this scoping review suggest that little research has investigated European citizens 75 years and older separately. The review found limited evidence of outcomes associated with usage of technology and level of education. Furthermore, issues of the accessibility of the devices were only partially covered in 1 of the included studies. Future research should cover these issues, as well as looking into what kind of social support is needed to facilitate the use of new telehealth systems among older citizens. As digital technology develops rapidly and life expectancy in Europe is expected to rise, additional and further research is required in order to investigate and estimate the future needs of older people.

## Data Availability

All data generated or analysed during this study are included in this published article, or in primary research articles and studies to which references were made.

## Abbreviations

WHO: World Health Organization.
ICT: information and communication technologies.
